# Feeding value of supplemental fat as a partial replacement for steam-flaked corn in diets for Holstein calves during the early growing phase

**DOI:** 10.1093/tas/txac048

**Published:** 2022-04-16

**Authors:** Alejandro Plascencia, Brooke C Latack, Pedro H V Carvalho, Richard A Zinn

**Affiliations:** 1 Departamento de Ciencias Naturales y Exactas, Universidad Autónoma de Occidente, Guasave, 81048, México; 2 Cooperative Extension, Division of Agriculture and Natural Resources, University of California, Holtville, CA 92250, USA; 3 Department of Animal Science, University of California, Davis, Davis, CA 95616, USA

**Keywords:** fat, feedlot, Holstein, performance, supplementation

## Abstract

In calf-fed Holstein steers, the early growing phase is characterized by very high relative dry matter intake (DMI) accompanied with lower-than-expected efficiency of energy utilization. Although fat is commonly supplemented in growing–finishing diets, the comparative feeding value has not been investigated during the initial receiving–growing period. Eighty-four Holstein calves (127.7 ± 2.1 kg body weight) were used to evaluate the effects of including 3.5% of supplemental yellow grease (YG) in the diet on characteristics of growth performance and dietary energy utilization of calves during the early growth phase. Morbidity and mortality were not affected by dietary treatments. Yellow grease supplementation did not affect DMI, but tended to increase average daily gain (4.6%; *P* = 0.07), and increased (*P* ≤ 0.03) gain efficiency (5.8%) and dietary net energy for maintenance (NE_m_) and gain (NE_g_) by 4.1% and 5.3%, respectively. Based on performance data, the estimated NE value of supplemental YG was 4.67 and 3.68 Mcal/kg for NE_m_ and NE_g_, respectively. These values are consistent with current tabular values assigned for vegetable oils (4.75 and 3.51 Mcal/kg), but markedly less (16.6%) than NE_m_ value assigned for YG (5.60 Mcal NEm/kg; NASEM, 2016. *Nutrient requirements of beef cattle*. 8th ed. Washington (DC): National Academy Press.).

## INTRODUCTION

The growth potential of calf-fed Holstein steers (live weight less than 275 kg at arrival) can be translated into gains at a rate of greater than 1% of their live weight during the initial 9-wk feedlot growing phase. Holstein steers have 8% to 10% greater feed intake than beef cattle (NASEM, 2016). Notwithstanding, their feed intake capacity during the early growing phase may not be sufficient to achieve maximal growth potential. Therefore, the energy density of diet at this phase is critical (Richeson et al., 2019). In this sense, fat supplementation is a common strategy to increase energy concentration the diet and hence, cattle growth performance. However, potential negative associative effects of supplemental fats (i.e., diet acceptability, reduced neutral detergent fiber digestion, among others; Hess et al., 2008) may detrimentally affect its feeding value. An important factor affecting the energy value of fat is the level of dietary fat intake. As level of total fat intake in the diet increases above 1.60 g/kg body weight (BW), the intestinal digestibility of fat begins to markedly decrease (Zinn, 1988; Plascencia et al., 2003; Zinn and Plascencia, 2004; Zinn and Jorquera, 2007). Thus, due to the comparatively high dry matter intake (DMI) of Holstein calves, the feeding value of fat may be reduced during the initial growing phase, even when supplemented at moderate levels (3.5%).

Another concern is the lower-than-expected efficiency of energy utilization of Holstein calves during the early growing phase. The basis for the lower-than-expected efficiency of energy utilization during early growing phase (first 112 d) appears to be largely the result of metabolizable amino acid deficiencies (Montaño et al., 2016; Torrentera et al., 2017). However, other factors may also be involved, as in some instances differences in metabolizable amino acid supplies did not fully explain differences in energetic efficiency (Salinas-Chavira et al., 2016; Carvalho et al., 2022). But be that as it may, decreased efficiency of energy utilization during the early growing phase may perhaps also affect the partial efficiency of energy utilization of supplemental fat. This is of particular interest due to the comparatively high cost of supplemental fats. Yellow grease (YG) is the most common form of recycled supplemental fat in the United States and México. Moreover, the feeding value of YG is comparable or even slightly greater than that of tallow (Plascencia and Zinn, 2019). To our knowledge, the comparative feeding value of supplemental fat has not been evaluated in Holstein calves during the initial early feedlot growing phase. Therefore, the objective of this experiment was to evaluate the effects of including 3.5% YG (DM basis) to the growing diets of Holstein calves during the early (first 9 wk) growing phase on growth performance, dietary energetics, and estimated energy value of supplemental YG.

## MATERIALS AND METHODS

The experiment was carried out in the Desert Research & Extension Center facilities of UC Davis located in Holtville, CA (32°48ʹ17.4ʹʹN 115°26ʹ49.3ʹʹW) 18 m below sea level with desertic climate. During the course of the experiment, temperature and relative humidity averaged 14.1 ± 3.0 °C and 49.6 ± 10.7 °C, respectively. Maximum temperature-humidity index averaged 67.2 ± 3.7. All animal care and handling techniques followed protocols approved by the University of California, Davis, Animal Use and Care Committee (#20548).

### Animal Processing, Housing, and Feeding

Eighty-four Holstein calves (127.7 ± 2.1 kg initial live weight) were used to evaluate treatment effects on characteristics of growth performance and dietary energetic of Holstein calves during the early (first 9 wk) growing phase. All calves in the study were previously castrated. Upon arrival, calves (approximately 4 mo of age) were vaccinated for bovine rhinotracheitis and parainfluenza 3 (TSV-27, SmithKline Beecham, West Chester, PA), clostridials (Fortress 7, Pfizer Animal Health, New York, NY), and Mannheimia haemolytica (One Shot, Pfizer Animal Health). Calves were treated against parasites (Ultramectin, R.X.V. Products, Kansas City, MO) and were injected with 1 × 10^6^ I.U. vitamin A (Vita-Jec A&D “500”, R.X.V. Products) branded, and ear-tagged. Calves were individually weighed off truck (initial shrunk weight; Hostetler Scales UMC555AAAAA, 5,000 lb × 1 lb, Imperial, CA) and randomly assigned to 14 pens (6 calves/pen). Pens were 5.48 × 9.14 m with 26.7 m^2^ of shade, and equipped with automatic waterers and fence-line feed bunks (4.27 m in length). Treatments consisted of a steam flaked corn-based diet supplemented with 0% or 3.5% YG (DM basis, Table 1). Supplemental YG was obtained from a recycling company (Baker Commodities Inc., Vernon, CA). Chemical compositions of YG which partially replace steam flaked corn in the control diet are shown as footnote in Table 1. Supplemental YG was added to the mixer prior to adding molasses (last added ingredient) in diet preparation. Steam-flaked corn was prepared as follows: A chest situated directly above the rollers (46 × 61 cm rolls, 5.5 corrugations/cm; Memco, Mills Rolls, Mill Engineering & Machinery Co., Oklahoma, CA) was filled to capacity (440 kg) with whole yellow corn (commercial blend of US #2 dent) and brought to a constant temperature (102 °C) at atmospheric pressure using steam (boiler pressure 60 psi). The corn was steamed for 20 min before starting the rollers. Approximately 440 kg of the initial steam-processed grain that exited the rolls during warm-up was not fed to steers on this study. Tension of the rollers was adjusted to provide the indicated flake density (0.31 or 0.36 kg/L). Flake density (kilograms/liter) was determined on freshly processed corn samples obtained directly beneath the rolls using a hand-held weight-per-bushel tester (Seedburo Equipment, Chicago, IL). Retention time of grain in the steam chamber was approximately 18 min. The steam-flaked corn was allowed to air dry (5 d) before use in diet preparation. Forages (sudangrass hay and alfalfa hay) were ground in a hammer mill (Bear Cat #1A-S, Westerns Land and Roller Co., Hastings, NE) with a 2.6-cm (sudangrass hay) or 5.0-cm (alfalfa hay) screen before incorporation into the complete mixed diets. Once all the ingredients were included in the mixer, the diets were mixed for 5 to 7 min. Diets were prepared at weekly intervals and stored in plywood boxes located in front of each pen. The experiment lasted 63 d. Calves were allowed ad libitum access to dietary treatments. New feed was provided twice daily at 0600 and 1400, offering approximately 30% of daily consumption in the morning feeding and the remainder in the afternoon feeding, allowing for a daily feed residual of ≈5%. Feed samples were collected from each batch and composited weekly for DM analysis (oven-drying at 105 °C until no further weight loss; method 930.15, Association of Official Analytical Chemists [AOAC], 2000) for determination of DMI.

**Table 1.** Composition of experimental diets (DM basis)

Item	Dietary treatment	
	0% YG	3.5% YG
Ingredient composition, % DM		
Sudangrass hay	8.00	8.00
Alfalfa hay	4.00	4.00
Yellow grease^1^	0.00	3.50
Molasses, cane	4.00	4.00
Steam flaked corn	72.39	68.89
Fishmeal	2.50	2.50
Canola meal	6.00	6.00
Urea	1.00	1.00
Limestone	1.55	1.55
Magnesium oxide	0.16	0.16
TM salt^2^	0.40	0.40
Dry matter, %	87.6	87.9
Calculated nutrients composition^3^		
NE_m_, Mcal/kg	2.07	2.20
NE_g_, Mcal/kg	1.42	1.53
Crude protein, %	15.56	15.27
Ether extract, %	3.85	7.17
Ash, %	6.17	6.10
NDF, %	15.36	15.05
Calcium, %	0.87	0.88
Phosphorus, %	0.41	0.41
Potassium, %	0.77	0.77
Magnesium, %	0.30	0.29
Sulfur, %	0.22	0.21

DM, dry matter; NDF, neutral detergent fiber; NE_g_, net energy for gain; NE_m_, net energy for maintenance; YG, yellow grease.

Chemical composition of yellow grease: total fatty acids, 93%; F.A.C. color 39; free fatty acids 15%; moisture, 2%, unsaponifiables, 2%, and insoluble material, 1%.

Trace mineral salt contained: CoSO_4_, 0.068%; CuSO_4_, 1.04%; FeSO_4_, 3.57%; ZnO, 0.75%; MnSO_4_, 1.07%; KI, 0.052%; and NaCl, 93.4%.

Based on nutritional values of each ingredient published by NASEM (2000).

### Estimation of Dietary Net Energy and Estimated Energy Value of Supplemental YG

Final live weight was multiplied by 0.96 to obtain estimated final shrunk BW (SBW; NASEM, 2000). Average daily gain (ADG) was determined as the difference in initial off-truck arrival SBW and final SBW divided by 63 (days on test). Gain efficiency (gain-to-feed ratio [G:F]) was determined as the ADG divided by corresponding DMI. Two approaches for evaluation of the efficiency of dietary energy utilization in growth performance trials are the observed-to-expected dietary net energy (NE) ratio and the observed-to-expected DMI ratio. Based on measures of growth performance (observed DMI, ADG, and average SBW), the observed dietary NE was calculated for each treatment by means of the quadratic formula according to the procedure of Zinn et al. (2008), as follows: *x* = (–*b* – √*b*^2^ – 4*ac*)/2*c*, where *x* = observed dietary NE_m_ (Mcal/kg), *a* = −0.41 EM, *b* = 0.877 EM + 0.41 DMI + EG, and *c* = −0.877 DMI; and EM = energy required for maintenance (NE_m_, Mcal/d) and EG = energy required for gain (NE_g_, Mcal/d) were estimated using equations published by NASEM (1984), as follows: EM = 0.077 × SBW^0.75^ and EG = ADG^1.097^ × 0.0557 BW^0.75^, and DMI correspond to the average daily DMI (kg) registered during the experiment.

The comparative NE_m_ value for the supplemental YG at 3.5% level of substitution was determined as follows: NE_m_ (Mcal/kg) of supplemental YG = [(NE_m_ observed for YG supplemented diet – NE_m_ observed for the control diet)/0.035] + 2.38. The divisor (0.035) represents the amount of supplemental YG in diet, and the 2.38 represents the NE_m_ value of steam flaked corn (NASEM, 2016) replaced by the supplemental YG. The NE_g_ value of tested YG was derived from its estimated NE_m_ value as follows: NE_g_, Mcal/kg = 0.877 NEm – 0.41 (Zinn et al., 2008).

### Statistical Design and Analysis

All data were analyzed as a completely random design experiment using Statistix 10 software (Analytical Software, Tallahassee, FL). Pens were used as experimental units. All the data were tested for normality using the Shapiro–Wilk test. In all cases, least squares means and standard error are reported and treatment effects were considered significant when the *P* value was ≤0.05 and tendencies are identified when the *P*-value was >0.05 and ≤ 0.10.

## RESULTS AND DISCUSSION

Quality characteristics of supplemental YG used in this study (Table 1) are consistent with the standards set by American Fats and Oils Association (AFOA, 2000). It is well known that fat composition, particularly total fatty acid content, is positively associated with its NE value (Zinn and Jorquera, 2007). Fatty acid concentration values equal to or greater than 90% are associated to the expected NE value of fats (6.03 Mcal NE_m_/kg), while fatty acid concentration values under 90% negatively affect its NE value, thus, the YG used here accomplished the quality grade to be considered “Good.”

Morbidity during the 9-wk feeding period was low (3.6%) and was not affected (*P* = 0.66) by dietary treatment (Table 2). There was no calf mortality. Historically, very low or no mortality during early growing phase of Holstein calves have been observed at this Research Center. This may be attributable, in part, to favorable environmental conditions consistent with the arid desert southwest in which calves were fed (Duff and McMurphy, 2007; Buda et al., 2020).

**Table 2.** Treatment effects on growth performance, dietary energy, and morbidity and mortality in growing Holstein steers receiving diet supplemented with 3.5% YG during early growth phase

	Supplemental yellow grease, %			
Item	0	3.5%	SEM	*P*-value
Pen replicates	7	7		
Days on fed	63	63		
Live weight (kg)				
Initial^1^	125.97	129.51	0.808	0.01
Final^2^	204.76	212.18	1.868	0.02
Daily gain (kg)	1.251	1.312	0.022	0.07
Dry matter intake (kg/d)	4.773	4.717	0.062	0.75
Gain to feed (kg/kg)	0.262	0.278	0.003	0.02
Observed dietary NE				
Maintenance	1.89	1.97	0.016	<0.01
Gain	1.25	1.32	0.014	<0.01
Observed-to-expected dietary NE				
Maintenance	0.91	0.90	0.008	0.23
Gain	0.88	0.86	0.010	0.28
Morbidity (%)	4.76	2.38	3.764	0.66
Mortality (%)	0.00	0.00	—	—

NE, net energy; YG, yellow grease.

Initial off-truck arrival weight.

Final shrunk weight (live weight reduced 4% to account for digestive tract fill).

Treatment effects on growth performance and dietary NE values are given in Table 2. Supplemental YG did not affect DMI (*P* = 0.77), but tended to increase ADG (4.6%; *P* = 0.07), and increased both gain efficiency (5.8%; *P* = 0.03) and dietary NE_m_ and NE_g_ (4.1% and 5.3%, respectively; *P* < 0.01). The relative DMI averaged 2.8% of BW. This value is in close agreement with the value of 2.73% projected for Holstein calves at similar weight consuming a 90:10 concentrate:roughage diet (Chester-Jones and DiCostanzo, 1996). Effects of YG supplementation on cattle DMI have been variable. Previous research has reported that inclusion of YG (from 3% up to 5%) in diets of calf-fed Holstein steers (Plascencia et al., 1999) and crossbreed cattle (DaSilva et al., 2019) did not affect DMI. However, Ramirez and Zinn (2000) and Plascencia and Zinn (2021) observed that YG supplementation decreased DMI of calf-fed Holsteins. Effects of supplemental YG on cattle DMI can be related to the level of fat supplementation, and composition and energy density of the diet (Hess et al., 2008). Based on results of the current study, YG supplemented at a moderate level (~3.5%) in high-energy diets was not expected to negatively affect DMI at this phase of growth (first 63 d on feed).

An important consideration for the addition of supplemental fat during the early growing phase is the potential effects on ADG that may carry through the subsequent growing–finishing phase. When there are no differences in DMI between control diet and YG supplemented diet, the replacement of corn in the diet with supplemental YG is expected to increase ADG and gain efficiency due to the enhanced dietary energy density (5.60 vs. 2.38 Mcal NE_m_/kg for YG and flaked corn, respectively; NASEM, 2016), and the positive associative effects of supplemental YG on ruminal fermentation, including inhibiting ruminal methane production (Zinn and Plascencia, 1996; Vargas et al., 2020). Dietary NE values in the current study were ~10% less than expected for both treatments (Table 2). In healthy cattle grown under non-stressful ambient conditions such as the current study, the ratio of observed-to-expected dietary NE would be 1.0. In other words, ADG is consistent with DMI and energy density of the diet. If the ratio is less than 1, energetic efficiency is less than expected. Lower efficiency of dietary energy utilization (lower observed-to-expected diet NE ratio) in crossbreed (Zinn and Shen, 1998; Montaño et al., 2016) and calf-fed Holstein calves (Zinn and Owens, 1993; Torrentera et al., 2017) is common during the initial growing period (first 112 d on feed on calf-fed Holsteins). This is largely attributable to dietary deficiencies of limiting essential amino acids during this early stage of growth (Torrentera et al., 2017). Based on observed DMI (4.75 kg) of the current experiment, metabolizable amino acid supply was expected to meet the theoretical requirements NASEM (2000, Level 1) for calves with average weight of 168 kg and ADG of 1.28 kg. Moreover, corn grain is a poor source of ruminal escape lysine, and the lysine content of corn can widely vary (NASEM, 2016; Arellano-Vázquez et al., 2017). Canola meal is a relatively poor source of metabolizable methionine. Accordingly, in order to meet theoretical requirements, diets were supplemented with 2.5% fishmeal, an excellent source of both metabolizable methionine and lysine. However, even though metabolizable amino acid supply was not the first limiting factor of the current experiment, efficiency of energy utilization was less than expected.

Based on performance data, the estimated NE value of the supplemental YG was 4.67 and 3.68 Mcal/kg for NE_m_ and NE_g_, respectively. These values are consistent with current tabular values assigned for vegetable oils (4.75 and 3.51 Mcal/kg; NASEM, 2000) but less than the values assigned for YG and tallow (5.6 and 4.43 Mcal/kg; NASEM, 2016). The digestibility (and energy value) of dietary fats is markedly affected by the fat intake (Zinn and Jorquera, 2007). The NE_m_ value of supplemental YG ranged from 6.02 to 6.35 Mcal/kg when total lipid intake did not exceed 1.60 g/kg BW (Zinn, 1988; Plascencia et al., 2003; Zinn and Plascencia, 2004). The estimated daily lipid intake of calves fed supplemental YG averaged 338 g in the current study. This corresponds approximately to 1.77 g of fatty acids/kg BW. Based on the partial efficiency of metabolizable energy utilization from dietary fat for BW gain (Plascencia et al., 2003), the expected NE_m_ value of YG is 5.43 Mcal NE_m_/kg, similar to the 5.60 expressed in current standards (NASEM, 2016). Accordingly, the expected NE value adjusted for the level of lipid intake is 14% greater than the observed NE value (4.67 Mcal NE_m_/kg) derived from calf growth performance. Based on the above, we tentatively speculate that when the overall efficiency of dietary energy utilization is less than expected (as is common during the early growing phase of calf-fed Holstein steers), and dietary fat intake is greater than 1.60 g/kg BW, the partial efficiency of utilization of supplemental fat will be likewise depressed.

## CONCLUSIONS

Inclusion of 3.5% supplemental YG in a steam flaked corn-based feedlot diet for Holstein calves did not detrimentally affect DMI, but tended to increase daily gain weight, and increased both gain efficiency and estimated dietary NE. Due to greater relative DMI of Holstein calves (2.8% BW), during the early growing phase, it is recommended that supplementation level does not exceed of 3.5% of DM in the diet.
